# Drivers of *Acacia* and *Eucalyptus* growth rate differ in strength and direction in restoration plantings across Australia

**DOI:** 10.1002/eap.2636

**Published:** 2022-06-02

**Authors:** Timothy L. Staples, Margaret M. Mayfield, Jacqueline R. England, John M. Dwyer

**Affiliations:** ^1^ School of Biological Sciences The University of Queensland Brisbane Queensland Australia; ^2^ CSIRO Land and Water, EcoSciences Precinct Dutton Park Queensland Australia; ^3^ CSIRO Land and Water Clayton South Victoria Australia

**Keywords:** Australian woodlands, density dependence, ecological restoration, forest planting, functional diversity, functional traits, maximum height, neighborhood diversity, specific leaf area, wood density

## Abstract

Functional traits are proxies for a species' ecology and physiology and are often correlated with plant vital rates. As such they have the potential to guide species selection for restoration projects. However, predictive trait‐based models often only explain a small proportion of plant performance, suggesting that commonly measured traits do not capture all important ecological differences between species. Some residual variation in vital rates may be evolutionarily conserved and captured using taxonomic groupings alongside common functional traits. We tested this hypothesis using growth rate data for 17,299 trees and shrubs from 80 species of *Eucalyptus* and 43 species of *Acacia*, two hyper‐diverse and co‐occurring genera, collected from 497 neighborhood plots in 137 Australian mixed‐species revegetation plantings. We modeled relative growth rates of individual plants as a function of environmental conditions, species‐mean functional traits, and neighbor density and diversity, across a moisture availability gradient. We then assessed whether the strength and direction of these relationships differed between the two genera. We found that the inclusion of genus‐specific relationships offered a significant but modest improvement to model fit (1.6%–1.7% greater *R* ^2^ than simpler models). More importantly, almost all correlates of growth rate differed between *Eucalyptus* and *Acacia* in strength, direction, or how they changed along the moisture gradient. These differences mapped onto physiological differences between the genera that were not captured solely by measured functional traits. Our findings suggest taxonomic groupings can capture or mediate variation in plant performance missed by common functional traits. The inclusion of taxonomy can provide a more nuanced understanding of how functional traits interact with abiotic and biotic conditions to drive plant performance, which may be important for constructing trait‐based frameworks to improve restoration outcomes.

## INTRODUCTION

Restoration of degraded ecological systems is a global priority (Suding et al., 2015)⁠, but designing successful ecological restoration remains a challenge (Brudvig et al., 2017; Suding, 2011)⁠. Success in ecological restoration is underpinned by plant survival and growth, and though plant growth rate is explicitly linked to the ecology and physiology of individual plants (Trenbath & Harper, 1978), generalizable approaches are needed to capture interspecific variation in plant growth in restoration settings. Plant functional traits can provide a common currency to compare ecological strategies and physiological tolerances across vastly different species because they are easily measured attributes that correlate with physiological processes linked to plant function (McGill et al., 2006; Violle et al., 2007). Functional traits correlate with several plant vital rates, including individual and population growth rate (Adler et al., 2014; Gibert et al., 2016; Poorter et al., 2008)⁠.

Comparative analyses of some plant functional traits have revealed global spectra describing the relationship between trait values and plant vital rates (e.g., Díaz et al., 2004)⁠. Wood density is consistently negatively correlated with diameter growth rate (Chave et al., 2009; Gibert et al., 2016)⁠, but species with dense wood tend to be more resistant to water stress, pathogens, and disturbance (Pérez‐Harguindeguy et al., 2013). In a similar vein, the leaf economic spectrum is underpinned by the tradeoff between leaf construction costs and photosynthetic capacity, which is captured in large part by specific leaf area (SLA) or its inverse, leaf mass per area (Díaz et al., 2016)⁠. High‐SLA species produce energetically cheap, short‐lived leaves that have a fast return on investment through photosynthesis (Reich, 2014; Reich et al., 1992), and SLA tends to correlate positively with plant growth rate (Wright & Westoby, 2000)⁠, especially in seedlings (Gibert et al., 2016)⁠. The maximum achievable height of a species is linked to plant strategies to intercept light while maintaining water transport and is a strong positive predictor of branch growth and overall growth rate (Gleason et al., 2018)⁠, but only in mature plants (Gibert et al., 2016)⁠. These three traits are not only easy to measure but are readily available for many species globally and load onto the two major axes of variation in global plant function (Sandra Díaz et al., 2016).

Functional traits link directly to resource acquisition strategies, and plant growth is mediated not only by trait values but by the degree to which they match local environmental conditions. At global scales, plant growth is driven by broad‐scale climatic gradients controlling the availability of resources, such as solar radiation, temperature, and precipitation (Huxman et al., 2004; Monsi et al., 2005; Rosenzweig, 1968)⁠. These conditions constrain viable combinations of plant traits that can survive and reproduce, acting as an environmental filter (Diaz et al., 1998; Messier et al., 2010). The global trait‐growth correlations described previously reflect this matching of environment and plant life history (Adler et al., 2014)⁠, but they are averages composed of substantial variation occurring at local scales (Messier et al., 2017; Zirbel & Brudvig, 2020)⁠. Even after filtering of nonviable trait combinations, local abiotic conditions can alter the magnitude, and even direction, of relationships between trait values and resulting plant growth rate (Thomas & Vesk, 2017; Zirbel et al., 2017)⁠, as can ontogeny (Gibert et al., 2016; Visser et al., 2016)⁠. Plant growth rate is also strongly influenced by the density, composition, and diversity of plants in the surrounding interaction neighborhood (Aerts, 1999)⁠. Competition is generally stronger among conspecifics than heterospecifics (Adler et al., 2018)⁠ because conspecifics share the same resource requirements and acquisition strategies (Abrams, 1983). In addition, the taxonomic and functional diversity of neighbors can influence plant growth rate by altering the diversity of surrounding and competing ecological strategies. Neighborhoods with greater functional diversity are predicted to have lower overlap in resource requirements and resource acquisition strategies (Loreau, 1998),⁠ leading to weaker competition between individuals and, consequently, faster growth rates (Chen et al., 2016; Zambrano et al., 2017).

Owing in part to this complex network of interacting factors, most trait‐based models only explain a small proportion of variation in plant growth rate (Paine et al., 2015; Yang et al., 2018). Among various sources of variation that are often ignored (e.g., the use of species mean trait values rather than individual‐level values [Swenson et al., 2020]), commonly measured functional traits may not capture all aspects of species' life‐history strategies that are relevant to a particular function (Li et al., 2017). There may be meaningful functional traits that have not been identified or known traits that are rarely included in such models (Worthy & Swenson, 2019). These missing traits may yet be captured by future research, and it is possible that new approaches will be developed to link easy‐to‐measure functional proxies to physiological traits that contribute to plant growth (Worthy & Swenson, 2019). In the meantime, restoration needs to improve planning frameworks and predictive ability to deliver better ecological outcomes at meaningfully large scales. While most studies of individual plant performance in restoration settings distinguish species by life form or successional stage (Gómez‐Aparicio, 2009; Verdú et al., 2012), frameworks have been developed to incorporate functional traits (Fiedler et al., 2018; Laughlin, 2014; Laughlin et al., 2018; Perring et al., 2015; Rosenfield & Müller, 2017)⁠. These frameworks will only succeed insofar as trait‐based models can reliably predict plant performance.

If some of the variation in plant growth rate not captured by functional traits is evolutionarily conserved, then incorporating phylogenetic information into trait‐based models may improve predictions (e.g., Li & Ives, 2017; but see Cadotte et al., 2017)⁠. Unfortunately, phylogenetic coverage of the plant kingdom is wide but lacks resolution at the species level (Hinchliff & Smith, 2014)⁠. Therefore, it may be infeasible to incorporate phylogenetics alongside functional traits in ecological restoration, which often involves diverse mixes of poorly studied species.

In this study, we tested the hypothesis that clear taxonomic groupings, such as genera, can capture evolutionarily conserved life‐history information that improves the predictive ability of trait‐growth models. Ideal genera to test this hypothesis are those that have

1. co‐occurring sets of species that are adapted to the same abiotic and biotic environments and generally experience the same growing conditions;
2. overlapping intergenus trait distributions, so that genera contain species with comparable ecological strategies; and
3. substantial evolutionary divergence, giving time for between‐genus life‐history variation to exceed within‐genus variation.

**FIGURE 1:**
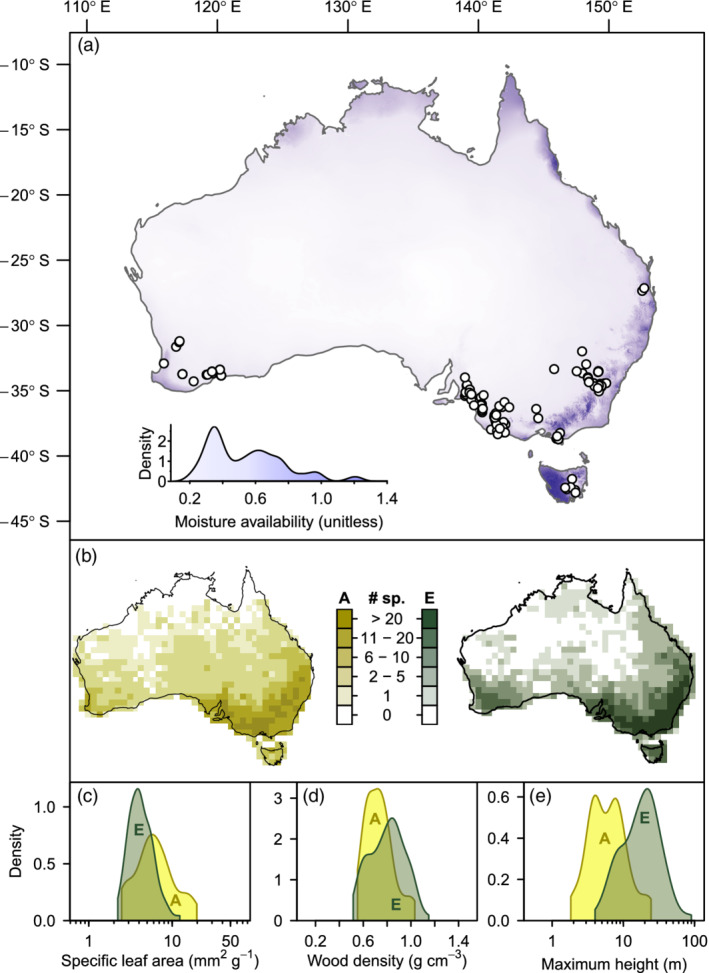
(a) Location of sampled mixed‐species reforestation plantings across Australia and their density across annual moisture availability gradient (unitless ratio of mean annual rainfall to mean annual potential evapotranspiration). Most plantings contained three sample plots (mean = 3.627, SD = 3.99) smaller than 400 m^2^, which were treated as interaction neighborhoods. (b) Number of *Acacia* (A) and *Eucalyptus* (E) species from sampled plantings (“# sp”) that occurred within 1° grid squares across Australia (43 and 80 species, respectively). Occurrence data were obtained from herbarium records (AVH, 2012). (c–e) Density of species mean trait values across sampled *Acacia* (A) and *Eucalyptus* (E) species. The widths of density functions were truncated at the most extreme trait values in our data set and indicate the width of intragenus trait variation compared to variation across all vascular plants (represented by plot width). The limits of (c) and (d) were set to the smallest and largest trait values used in large‐scale studies: Wright et al. (2004) for specific leaf area (converted from leaf mass per area) and Chave et al. (2009) for wood density. Limits for maximum height (e) were set to 0.1 and 110 m.

*Acacia* and *Eucalyptus*, two common Australian woody plant genera, satisfy all three of these requirements and in addition are important to restoration efforts across Australia and to forestry globally. These two genera are members of different plant orders (*Fabales* and *Myrtales*, respectively), diverging ~106 million years ago (Kumar et al., 2017)⁠. Both are extremely diverse (more than 1000 and 700 recognized species in *Acacia* and *Eucalyptus*, respectively), having co‐occurring species across most of their distribution (Figure 1b) with wide and largely overlapping intragenus variation in common functional traits (Figure 1c–e).

Here we analyzed inventory data for 10,378 *Eucalyptus* individuals from 80 species and 6921 *Acacia* individuals from 43 species, measured in 497 neighborhood plots in 137 diverse reforestation plantings across southern Australia (Figure 1a). Focusing on individual plant growth rate in these mixed‐species plantings, we posed the following questions:

Does adding genus‐specific relationships between growth rate and trait, environment and neighborhood variables improve model performance?
(a) How do functional traits and environmental and neighborhood correlations with plant growth rate differ between the two genera? And (b) do these relationships vary across a large moisture availability gradient?

Predictors of growth rate included in our study, as well as their theorized relationship with growth rate, are summarized in Table 1.

**TABLE 1:** Continuous fixed effects included in models of plant growth rate, with theorized relationship with growth rate and expected correlation direction

Fixed effect	Theoretical link to growth rate	Direction of relationship	Example
Focal plant functional traits			
Specific leaf area	Leaf economics spectrum	Positive	Wright et al. (2004)
Wood density	Wood economics spectrum	Negative	Chave et al. (2009)⁠
Maximum height	Allocation to vegetative growth	Positive	Stephenson et al. (2014)⁠
Climate			
Moisture availability	Resource for energy production	Positive	Huxman et al. (2004)⁠
Solar radiation	Resource for energy production	Positive	Monsi et al. (2005) ⁠
Planting characteristics			
Planting age	Plant growth curves	Negative	Paine et al. (2012)⁠
Plot area	Control for varying plot sizes	…	…
Neighborhood characteristics			
Neighbor density	Competition for limiting resources	Negative	Tilman (1987)⁠
Relative abundance of intraspecific neighbors	Negative density dependence	Negative	Johnson et al. (2012)⁠
Species richness	Complementarity	Positive	Loreau (1998)⁠
Functional evenness	Complementarity	Positive	Loreau (1998)⁠
Functional dispersion	Complementarity	Positive	Loreau (1998)⁠

## METHODS

### Restoration plot data

Neighborhood plots used in this study were set up in environmental plantings across the arable region of southern Australia (Figure 1a). Survey data were collected by the Commonwealth Scientific and Industrial Research Organization (CSIRO) (17,081 plants in 226 plots) and not‐for‐profit organizations: Greening Australia (9902 plants in 221 plots) and Greenfleet (1046 plants in 50 plots). These environmental plantings were established on ex‐agricultural land using multiple native species planted as tubestock. In all planting locations, the agricultural land had long been cleared of forest or woodland vegetation. Some study plantings were established expressly for ecological restoration, others for alternativee purposes such as carbon sequestration. Mean planting age was 12 years (σ = 5.27), but ages ranged from 4 to 33 years.

The study plantings spanned a wide moisture availability gradient across southern Australia from semiarid to mesic conditions (Figure 1a). Natural vegetation in this region varies from open woodlands with 8‐ to 10‐m canopies and an understory shrub layer to tall, closed forests with 30‐m canopies. In mesic regions, some tree species can extend well beyond 50 m tall (e.g., *Eucalyptus regnans*). Across the breadth of our study region, *Eucalyptus* and *Acacia* species commonly codominate the canopy and subcanopy layers, respectively. *Eucalyptus* species range from 10‐m‐tall multistemmed “mallees” to 90‐m‐tall trees (Brooker & Kleinig, 2006), while *Acacia* are nitrogen fixers that grow from 0.5‐m‐tall spreading shrubs to 25‐m‐tall trees (Maslin et al., 2003). Though our study species only comprised around 5% of *Acacia* species and around 10% of *Eucalyptus* species, their combined geographic distribution covers much of Australia and overlaps considerably (Figure 1b).

Neighborhood plots within restoration plantings varied in size, but all were squares smaller than 400 m^2^. Plots contained an average of 34.81 woody plants (σ = 25.61) from 4.00 species (σ = 1.84) and had an average area of 231.84 m^2^ (σ = 115.68 m). In total, plots contained stem diameter measurements on 28,029 individual plants from 249 species and 38 genera. In each plot, we treated all *Acacia* and *Eucalyptus* plants with available species‐level functional trait data as focal plants for growth rate analyses (6921 *Acacia* plants from 43 species and 10,378 *Eucalyptus* plants from 80 species).

### Biomass estimation

Diameter measures on all stems within each plot were taken during plot surveys, either at breast height (130 cm above the ground) or 50 or 10 cm above the ground. Aboveground biomass of each plant was estimated from stem diameter measurements using species‐ or life‐form‐specific allometric equations (as per Paul et al. [2013]). These equations were validated using plants harvested for biomass and had very low levels of error (Paul et al., 2013)⁠.

### Functional traits

We included three plant functional traits in our analyses, estimated as mean values for each species: SLA (mm^2^ g^−1^), wood density (g cm^−3^), and maximum height (m) (see Appendix S1: Table S1 for trait sample sizes). The majority of functional trait data were obtained from the TRY plant database (Fonseca et al., 2000; Green, 2009; Kattge et al., 2009; Niinemets, 2001; Onoda et al., 2011; Shipley et al., 2006; Wirth & Lichstein, 2009) and Australian state herbarium records. We collected additional trait data from naturally growing individuals for this study, for 65 and 63 species for SLA and wood density, respectively (Appendix S1: Table S2). These traits were measured on five individuals (five leaves per individual for SLA, one stem sample per individual for wood density), following standard trait‐collection protocols (Pérez‐Harguindeguy et al., 2013).

Where possible, we obtained traits for all neighboring species (not just focal *Acacia* and *Eucalyptus* species). For each nonfocal species with unknown trait values, we interpolated trait values using the mean trait value of all congeners from nearby plots (within 50 km for ~90% of neighbors with missing trait data). Of the 126 nonfocal species, we had species mean traits for 46.1% for SLA, 50.8% for wood density, and 81.7% for maximum height. After interpolation, we had estimated values for 94.4% for SLA, 93.6% for wood density, and 96.8% for maximum height. Focal *Eucalyptus* and *Acacia* species did not contain interpolated trait values; we had species mean traits for all 123 species.

### Data analysis

All analyses were performed in R (R Core Team, 2021).

#### Calculating growth rate

We estimated relative growth rate for each focal plant by dividing its estimated aboveground biomass (log‐normal transformed) by planting age. The resulting growth rate is relative annual biomass increment, in units of kilograms per kilogram per year. This calculation assumes that a plant's initial mass at the time of planting was zero and that all surveyed plants were present from time of planting. Because plots were not inventoried immediately following planting, it is possible that some may have subsequently colonized via natural means. As a precaution, we excluded very small focal plants (<100 g aboveground biomass) from our analyses because they were unlikely to have been present at the initial planting (*n* = 129).

### Model predictors

We extracted planting‐level estimates of climate variables from spatial layers using planting longitude and latitude coordinates (extract function, raster package [Hijmans, 2021]⁠). We extracted estimates of mean monthly solar radiation (in megajoules per square meter per day) for each planting from Gallant et al. (2014), which used a Digital Elevation Model informed by climate, albedo, and vegetation cover observations. We averaged long‐term monthly mean solar radiation values for each planting into a single long‐term annual mean.

We combined estimates of long‐term annual precipitation and potential evapotranspiration (PET) into a ratio of moisture availability (unitless: annual rainfall/annual potential evapotranspiration, such that higher values indicate greater available moisture). We obtained annual precipitation (mm) estimates from Hijmans et al. (2005), who used 1950–2000 global weather station records to interpolate across a 30″ spatial layer that forms part of the WorldClim Global Climate Data product. We extracted PET estimates from Trabucco and Zomer (2009), who used WorldClim data to calculate PET using the Penman–Montieth reference evapotranspiration equation.

We relied on solar radiation and moisture availability to represent each planting's abiotic environment for three reasons. First, these variables directly reflect access to resources needed for plant growth (Table 1). Second, they correlate strongly with other important climatic constraints on plant growth, such as maximum temperature. Finally, plantings occurred across a spectrum of both variables that allowed us to model not only their direct correlation with plant growth but also their interaction.

As well as the two abiotic variables, we included log‐normal transformed planting age (in years) as a separate predictor variable in our growth models, in addition to its use in estimating focal plant relative growth rate. Planting age reflects the age of focal plants and captures the deceleration of relative growth rate as plants age (Paine et al., 2012)⁠. We also included plot area as a covariate to capture whether growth rate differed systematically in small versus large neighborhood plots.

Stem locations were not mapped within plots, so it was not possible to estimate neighborhood conditions using a specific radius around each *Eucalyptus* and *Acacia* focal plant. Instead, we calculated neighborhood attributes using all plants in the plot (not just *Acacia* and *Eucalyptus*), excluding the focal, and accounted for differences in plot size and neighbor density statistically. We calculated neighbor density (plants per hectare) by dividing the number of plants in the plot (minus the focal) by plot area (in hectares). We estimated neighborhood richness using rarefaction, to account for variation in plot size and neighbor abundance. Rarefied richness was calculated using the rarefy function in the vegan package (*n* = 9: minimum of 10 plants per plot, minus the focal) (Oksanen et al., 2020). We also calculated functional diversity indices for each neighborhood using the three highlighted functional traits: SLA, wood density, and maximum height. We calculated functional evenness (Villéger et al., 2008) and functional dispersion (Laliberte & Legendre, 2010) of the neighborhood around each focal using the dbFD function in the FD package (Laliberté & Shipley, 2011). Functional evenness captures the spacing of species in trait space (Villéger et al., 2008). High‐evenness neighborhoods contain species with growth strategies spread evenly across occupied trait space. Low‐evenness neighborhoods contain clusters of growth strategies, with unoccupied gaps in trait space. Functional dispersion represents the spread of functional strategies relative to the abundance‐weighted centroid of trait space (Laliberte & Legendre, 2010). Low‐divergence neighborhoods contain species with very similar functional traits (i.e., species points are close to the centroid in trait space). High‐divergence neighborhoods contain species with very different strategies (i.e., species points tend to occur away from the centroid and at the edges of occupied trait space). We also calculated functional range (as per Villéger et al. [2008]), but it was highly correlated with species richness (*r* = 0.77). We excluded functional range and retained species richness as our estimate of the diversity of growth strategies in our neighborhood plots.

### Statistical models of plant growth rates

#### Models using observed growth rates

We modeled relative growth rates using linear mixed‐effects models (lmer function, lme4 package [Bates et al., 2015]), using restricted maximum likelihood. Random effects (varying intercepts) were included to account for nesting of focal plants within sample plots, plots within plantings, and plantings within biogeographic subregions (version 7 of the Interim Biogeographical Regionalisation of Australia [IBRA] [Thackway & Cresswell, 1995]). We also included focal species as a separate random effect to account for the fact that growth rate observations of focal plants from the same species were not independent. The species random effect was crossed with the spatial random effects because many focal species were planted in multiple plantings.

We log‐normal transformed three predictor variables—planting age, maximum height, and solar radiation—and square‐root transformed neighbor density prior to modeling to improve the linearity of bivariate relationships with relative growth rate. All predictor variables were standardized (μ = 0, σ = 1) before modeling to allow for direct comparison of partial regression slopes. Despite some correlations between predictor variables, variance inflation factors for all predictors were less than three. Finally, because plot area was a covariate to account for nonstandardized plot sizes, we initially allowed all other predictors to interact with plot area. Only the proportion of intraspecific neighbors was significant; this interaction was retained in all relevant models in what follows.

Within this structure we used seven models of increasing complexity to address our research questions. Our first model included only an intercept term (base model). Our second model included focal species' traits, climate variables, planting characteristics, and neighborhood metrics (Table 1) but did not include focal plant genus (no genus model). Our third model added the focal plant genus (*Acacia* and *Eucalyptus*) to the second model as a separate predictor, fitting separate intercepts to each genus (genus intercepts model). The fourth model retained separate genus intercepts and added two‐way interactions between genus and the other predictors (genus slopes model).

The final three models extended our no genus, genus intercepts, and genus slopes models by including interactions between moisture availability and all other predictors. For the models extending our no genus and genus intercepts models, these were two‐way continuous interactions (no genus moisture model and genus intercepts moisture model). For the model extending our genus slope model (genus slopes moisture model), these were three‐way interactions between moisture availability, genus identity, and each other continuous variable.

We compared our models using the Akaike information criterion (AIC) (Akaike, 1974)⁠ and chi‐square (*χ* ^2^) likelihood ratio tests. Models were refit using maximum likelihood prior to likelihood comparison. We also calculated marginal and conditional pseudo‐*R* ^2^ values to estimate the proportion of growth rate variance explained by each model (as per Nakagawa & Schielzeth [2013]).

### Models establishing null distributions of growth rate relationships

To minimize the influence of multiple focal observations and uncertainty of focal locations within each study plot, we used a randomization approach to establish null expectations for growth rate correlations (similar to Mitchell‐Olds, 1987; Dwyer et al., 2010). We generated null expectations for two of our models (genus slopes and genus moisture slopes models) by randomly shuffling growth rates between focal trees across our data set 1000 times. We fit each of these random data sets using the model structure of the genus slopes and genus slopes moisture models and extracted standardized slope estimates from each to generate null slope distributions (which were centered at zero). We compared observed growth rate models to the distribution of null slopes by calculating the probability that the observed partial regression slope (holding other predictors at their mean) fell within the null slope distributions. For the genus slopes moisture model, these partial regression slope comparisons were made at the 10th and 90th quantiles of moisture availability in our data set. This corresponds to moisture availability values of 0.27 and 0.76, which we refer to as dry and mesic conditions, respectively.

## RESULTS

**FIGURE 2:**
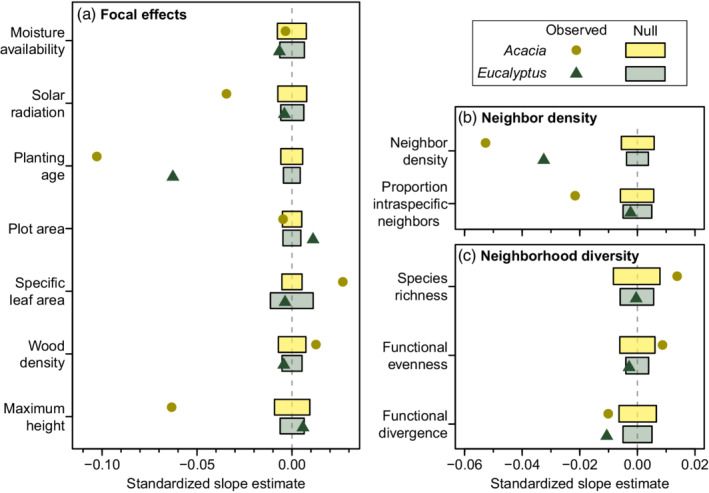
Slope estimates for climate, focal plant traits, neighborhood density, and neighborhood diversity variables from models of growth rate for *Eucalyptus* and *Acacia* focal plants. Points are standardized partial slopes from models fitted to all focal plants (“Observed”). Rectangles represent the 95% confidence intervals of null distributions of slopes from 1000 models where growth rates were randomly swapped between focal plants (“Null”). Where observed slope estimates do not overlap with this space, observed slopes were considered to be different from zero (Appendix S1: Table S8).

**FIGURE 3:**
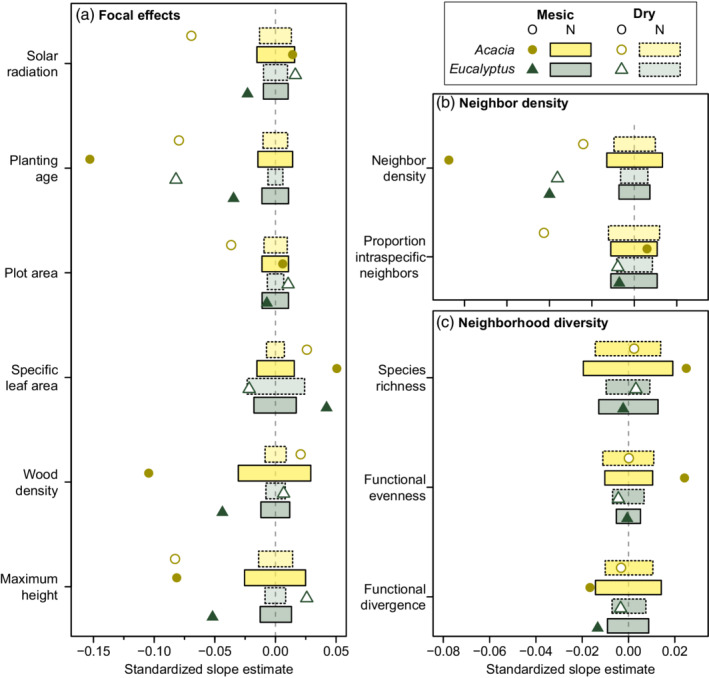
Slope estimates for interactions between moisture availability and other fixed effects on *Eucalyptus* and *Acacia* growth rate. Points represent observed slope estimates (“Observed”) in dry and mesic locations, predicted using 10th and 90th quantiles of moisture availability in our study data (0.27 and 0.76, respectively, where a value of 1 represents a balance between mean annual rainfall and mean annual potential evapotranspiration). Rectangles represent the 95% confidence intervals of null distributions of slopes (“Null”) from 1000 models where growth rates were randomly swapped between focal plants. Solid rectangles represent confidence intervals in mesic conditions, dashed rectangles in dry conditions. Where observed slope estimates do not overlap with these null spaces, observed slopes were likely to be different from zero (Appendix S1: Table S9). Partial regression slopes for these coefficients on raw predictor scales are shown in Appendix S1: Figure S1.

We found genus‐specific intercept and slope terms improved the fit of plant growth rate models, albeit with only modest increases in explained variation in plant growth rate (Table 2: Question 1). *Acacia* and *Eucalyptus* growth rate correlations differed in either strength or direction for almost all predictors, including measured functional traits, climate variables, and neighborhood conditions (Figure 2: Question 2a). In addition, a number of these genus‐specific correlations changed in strength or direction across the sampled moisture gradient (Figure 3: Question 2b).

### Does inclusion of genus‐specific terms improve growth model performance?

Including only separate intercepts for *Acacia* and *Eucalyptus* did not substantially improve model fit over equivalent no genus models, but the inclusion of genus‐specific slopes did (Table 2). Likelihood ratio tests between the no genus model and genus intercepts model (*χ* ^2^ = 3.200, *p* = 0.073) and between the no genus moisture model and genus intercepts moisture model (*χ* ^2^ = 3.781, *p* = 0.151) were not significant. The inclusion of genus‐specific slopes significantly improved model fit compared to the corresponding no genus model (no genus vs. genus slopes *χ* ^2^ = 265.318, *p* = <0.001: no genus moisture vs. genus slopes moisture *χ* ^2^ = 515.094, *p* = <0.001), explaining 1.60% and 1.70% more variation in growth rate, respectively (Table 2). The addition of three‐way interactions in the genus slopes moisture model explained more variance than simpler two‐way interactions in the genus slopes model (Table 2, *χ* ^2^ = 397.880, *p* < 0.001). This more complex model identified a number of growth rate relationships that were modulated by both genus identity and moisture availability (Figure 3).

**TABLE 2:** Comparison of models of *Eucalyptus* and *Acacia* growth (all focal plants)

Model name	AIC	ΔAIC from			Pseudo‐*R* ^2^	
		Base model	Genus intercepts model	No genus moisture model	Marginal	Conditional
Base model	−17,116.76	…	…	…	0.000	0.544
No genus model	−17,223.28	−106.52	…	…	0.174	0.540
Genus intercepts model	−17,218.96	−102.20	…	…	0.167	0.530
Genus slopes model	−17,345.33	−228.57	…	…	0.190	0.530
No genus moisture model	−17,234.75	−117.99	…	…	0.200	0.544
Genus intercepts moisture model	−17,222.35	−105.59	122.98	12.40	0.192	0.537
Genus slopes moisture model	−17,481.06	−364.30	−135.73	−246.31	0.217	0.547

*Note*: The base model contained a single, global, intercept term. No genus models contained the additional predictors outlined in Table 1. The genus intercepts model included these predictors alongside a two‐level genus factor (*Acacia* vs. *Eucalyptus*), fitting separate intercepts for each genus. The genus slopes model fitted separate intercepts as well as interactions between genus and the other predictors. Extending on these models, we fit three additional models with interactions between moisture availability and all other predictors. Slope estimates for each predictor variable in the genus slopes model and genus slopes moisture model are illustrated in Figures 2 and 3, respectively. Abbreviation: AIC, Akaike information criterion; ΔAIC, change in Akaike information criterion.

### Correlation differences between genera and across moisture gradient

We found a number of nonzero correlations between plant growth rates and predictor variables for *Acacia* and *Eucalyptus* (Figure 2; Appendix S1: Tables S5 and S8). Without considering genus‐specific intercepts or slopes, functional trait, environmental, and local neighborhood correlations explained 17.4% of the variation in plant relative growth rate (no genus model: Table 2).

#### Planting conditions, age, and plot size

Moisture availability and solar radiation were mostly weak predictors of plant growth rate (Figure 2). The one exception to this was the negative correlation between *Acacia* growth rate and solar radiation (Figure 2a; Appendix S1: Tables S5 and S8). Adding interactions with moisture availability affected solar radiation slopes for both genera, but in different ways. Increased solar radiation slowed *Acacia* growth rate in dry conditions but had no effect in mesic regions (Figure 3a; Appendix S1: Tables S7 and S9). In contrast, increased solar radiation improved *Eucalyptus* growth rate in dry regions (although weaker than the negative *Acacia* slope) but slowed growth rate in mesic conditions (Figure 3a; Appendix S1: Tables S7 and S9).

Growth rate in older plantings was consistently slower for both genera (Figure 2a), but moisture availability modulated this in different ways. *Acacia*'s negative relationship between growth rate and planting age grew stronger as conditions became more mesic, whereas *Eucalyptus*' relationship weakened (Figure 3a; Appendix S1: Tables S7 and S9). Plot area was included in the models to correct for varying plot sizes and was not a mechanistic variable. We found that *Acacia* growth rate was slower in smaller, dry plots, and *Eucalyptus* growth rate was slightly faster (Figure 3a; Appendix S1: Tables S7 and S9). We observed no effect in more mesic plots for either genus (Figure 3; Appendix S1: Tables S7 and S9).

#### Focal plant functional traits

All three functional traits correlated with growth rate. In *Acacia* plants, the genus slopes model predicted faster growth in shorter *Acacia* species, species with higher SLA, and species with higher wood density. These trait–growth relationships were largely consistent across the moisture gradient, except for wood density. Higher wood density shifted from providing a growth advantage for *Acacia* in dry conditions to slowing growth in mesic conditions (Figure 3a; Appendix S1: Tables S7 and S9). The positive *Acacia* SLA slope became somewhat stronger in mesic conditions, but the negative effect of maximum height was almost identical (Figure 3a; Appendix S1: Tables S7 and S9).

In contrast, only maximum height showed a nonzero correlation with *Eucalyptus* growth rate in the genus slopes model; growth rate increased only in taller *Eucalyptus* species, and this slope was only marginally significant compared to null expectations (*p* = 0.039). Including interactions with moisture availability revealed that *Eucalyptus*' near‐zero slopes were averages of relationships that differed based on moisture (Figure 3; Appendix S1: Tables S7 and S9). In mesic conditions, shorter *Eucalyptus* species with higher SLA and lower wood density grew faster (Figure 3; Appendix S1: Tables S7 and S9). In dry conditions, the only significant effect was with maximum height, where taller *Eucalyptus* species grew faster.

#### Neighborhood density and diversity

Plants from both genera exhibited slower growth as neighbor density increased (Figure 2b; Appendix S1: Tables S5 and S8). *Acacia* also exhibited signs of negative density dependence towards conspecifics, with a significant negative correlation between growth rate and the proportion of intraspecific neighbors (Figure 2b; Appendix S1: Tables S5 and S8). The effect of neighbor density on *Eucalyptus* growth rate was consistent across dry and mesic conditions (Figure 3b; Appendix S1: Tables S7 and S9). *Acacia* growth rate was more strongly affected by neighbor density in mesic conditions, although still negatively impacted in dry conditions (Figure 3b; Appendix S1: Tables S7 and S9). The observed negative density dependence in *Acacia* was only present in drier conditions (Figure 3b; Appendix S1: Tables S7 and S9).

*Acacia* grew faster in more diverse and functionally even neighborhoods (Figure 2c; Appendix S1: Tables S5 and S8). Both genera exhibited slower growth rates in neighborhoods with less dispersion in growth strategies (Figure 2c; Appendix S1: Tables S5 and S8). When we added interaction effects involving moisture availability, these significant diversity relationships only persisted in mesic conditions, with zero correlations in dry conditions (Figure 3c; Appendix S1: Tables S7 and S9).

## DISCUSSION

We identified consistently divergent growth rate correlations across 40 *Acacia* and 83 *Eucalyptus* species and along a large moisture availability gradient. Species of these two genera commonly co‐occur and have largely overlapping trait distributions, but only one growth rate correlation was coordinated across the two genera when moisture availability was considered: a small reduction in growth rate in neighborhoods with low functional divergence. Functional ecology is grappling with the low predictive ability of species‐mean functional traits in trait‐based models (Paine et al., 2015) while also promoting the application of functional traits to restoration ecology (Perring et al., 2015). While the inclusion of genus‐specific slopes provided only modest improvements in model fit relative to simpler models, they clarified the context dependence of trait and neighborhood relationships with plant growth. Restoration trait frameworks use the relationship between traits, plant performance, and environment (often under the “response” and “effect” trait framework [proposed by Lavorel & Garnier, 2002]) to design restoration assemblages for a target outcome (Laughlin, 2014; Laughlin et al., 2018). In the absence of high‐resolution phylogenetic data, our results suggest taxonomic groupings can improve trait‐based models of plant performance and offer tangible benefits to restoration design frameworks.

### Comparison of *Acacia* and *Eucalyptus* growth rate correlates

In mesic conditions, we observed growth rate correlations in both *Acacia* and *Eucalyptus* that accorded with expectations from global trait–growth syntheses. Growth rate was greater in species with higher SLA and lower wood density, in accordance with the leaf (Wright et al., 2004) and wood economic spectra (Chave et al., 2009). We also found slower growth in taller species, which likely reflects the immaturity of many plants in our study plantings (maximum age since planting was 33 years, with a mean of 12 years); positive height–growth rate correlations are typically only evident in mature trees (Gibert et al., 2016). The accordance with global trait–growth relationships observed in mesic conditions broke down in drier areas of our study region. The direction of growth correlations for all three functional traits changed across our moisture gradient in *Eucalyptus* but shifted only for wood density in *Acacia*. These divergent correlations map to differences in moisture stress adaptations between *Acacia* and *Eucalyptus*, in both below‐ and aboveground structure.

Faster growth in high‐wood‐density species, as observed in *Acacia*, is possible in regions with seasonally dry periods. High wood density allows for continual growth under conditions where softer‐wood species have shut down water transport, and therefore growth, to avoid xylem collapse (Chave et al., 2009; O'Grady et al., 2009)⁠. This positive influence of wood density on growth may be limited; at some point, solar radiation and low soil moisture will likely reduce *Acacia* growth despite wood density (Phillips & Riha, 1993; Yates et al., 2000). The negative relationship between *Acacia* growth rate and solar radiation (which correlated with moisture availability: *r* = −0.69) in dry regions may reflect this limitation. In drier plantings, all *Acacia* species exhibited a moisture‐stress‐induced slowdown of growth, but higher‐wood‐density species were less affected than softer‐wood species. Wood density may have less of a buffering effect on *Eucalyptus* growth in dry regions, hence the zero correlation, because many species can grow deep root systems to access groundwater (O'Grady et al., 1999)⁠. This may only be a feasible strategy for *Eucalyptus* in regions with shorter dry periods and accessible water tables (O'Grady et al., 2009), which partially explain the reduced distribution of *Eucalyptus* species in more arid regions of Australia (central and northwest Australia: Figure 1a; distribution of *Eucalyptus* species included in our study shown in Figure 1c). This explanation may extend to the differing correlations between growth rate and solar radiation across the two genera, likely associated with SLA–growth rate correlations. *Acacia*, which had a consistently positive SLA–growth rate correlation, showed decreased growth with more solar radiation in dry regions, where moisture limitation may reduce the benefits of higher‐SLA leaves to growth rate. *Eucalyptus* showed this pattern in mesic conditions, but not in dry conditions. This may reflect *Eucalyptus* leaf traits that minimize periods of nonfunctionality in regions with severe moisture limitation, rather than traits that maximize growth rate (Rosas et al., 2021).⁠

### Genus‐based response to neighborhood conditions

We found that *Acacia* growth rate was sensitive to neighbor density, especially compared to *Eucalyptus*. *Acacia* plants surrounded by conspecific neighbors were also smaller than expected for their age (Appendix S1: Table S10). Both of these patterns aligned with expectations from limiting similarity and negative density‐dependent plant growth (Adler et al., 2018; Wilson, 2007)⁠. In contrast, *Eucalyptus* growth rate was not affected by conspecific neighbors or by neighborhood diversity outside a small growth reduction in less divergent neighborhoods. This may be because *Eucalyptus* species are more tolerant to competition, especially intraspecific competition, while immature. Alternatively, the negative competitive effect exerted by conspecific neighbors may have been balanced by excluding heterospecifics via allelopathy (Zhang & Fu, 2009)⁠. While not quantified in our study, grass cover can strongly suppress woody plant growth in young reforestation (Pinto et al., 2015; Standish et al., 2008). If this were the case, the balance between facilitation (via grass suppression) and competition of intraspecific neighbors would likely deteriorate as *Eucalyptus* trees matured, where overlapping crown and root zones would increase competitive intensity.

We expected planting age to capture the near universal slowdown of relative growth rate that occurs as plants grow (Paine et al., 2012). We observed this for both genera in mesic and dry conditions. The faster reduction in older *Acacia* plants in mesic conditions may be linked to an increase in the size of neighbors. Our neighbor density variable only quantified the number of neighbors, not their size. In mesic conditions, neighbors may have been larger and exerted a greater competitive effect on *Acacia* focal plants.

### Growth rate, plant performance, and limitations

It is important to note that our study focused wholly on aboveground growth rate, to the exclusion of other aspects of plant performance, such as survival, biomass allocation to root mass, or reproductive output. Our metrics of growth rate are also calculated from a single time comparison; they do not capture biomass lost to herbivory, leaf litter, or mechanical damage. Some of the negative growth rate correlations we observed do not necessarily mean species have maladaptive trait values for the given environment. Instead, they may represent tradeoffs between growth rate and survival, where the trait value (or trait combination) that increases survival under given conditions also decreases growth rate (Chen et al., 2019). An example may be the slow growth rate in *Acacia* species in dry regions with high solar radiation. Alternatively, plant growth may be consistent under dry conditions, but a greater fraction was allocated to root biomass. Indeed, *Acacia* has been shown to invest more in root biomass when moisture‐stressed than under favorable conditions (Forster et al., 2016).

Most of the residual variation in growth rate was within our smallest random‐effect group, which consisted of individual focal plants within each neighborhood plot. This variation could have resulted from microclimate environmental variation or nonwoody competition (e.g., grasses), but it could also have arisen from our use of species mean trait values and the lack of spatial coordinates for each plant. Species mean trait values are strongly correlated with plant performance, as observed in our results, but intraspecific variation is important, especially when considering how trait values map onto environmental conditions across a species' range or even across local‐scale environmental gradients (Dwyer et al., 2014). Differences in variation or plasticity of trait values between species may have implications for growth rate and adaptive potential in different environments (e.g., Pohlman et al., 2005). The spatial arrangement of plants within each study plot would have allowed us to more accurately quantify each focal plant's interaction neighborhood and permitted the calculation of trait hierarchies and distances between focal plants and neighbors, which have proven effective as predictors of growth rate (Kunstler et al., 2016; Uriarte et al., 2010)⁠.

Our results here reflect correlation to past climatic conditions and to long‐term climate averages. We highlight how variable trait–growth and neighborhood–growth relationships can be, both between genera and across spatial environmental gradients. However, changes under climate warming scenarios have the potential to alter environmental filters that have acted on local communities, potentially making previously successful trait combinations unviable. This is a prime concern in ecological restoration, where consensus is shifting from restoring past ecosystem states to restoring and managing ecosystem functions (Harris et al., 2006; Simonson et al., 2021)⁠. Southern Australia is predicted to become more susceptible to drought under climate change (IPCC, 2021)⁠, potentially resulting in a reduction in soil moisture available to plants. While our models are space‐for‐time substitutions, and caution must be taken generalizing our conclusions to the growth of *Acacia* and *Eucalyptus* plants in future restoration settings, we may expect growth correlations in mesic regions to shift toward patterns we observed in drier regions (as per Figure 3). Plantings already in dry regions, expected to become drier over time, extend beyond the bounds of conditions sampled in our study. Our results accord with other work emphasizing that understanding the impacts of changing conditions, and resulting plant performance, will be essential to restoration success in the future (Brudvig, 2017; Zirbel et al., 2017; Zirbel & Brudvig, 2020)⁠.

## CONCLUSIONS

Our models explained a substantial proportion of individual plant growth for many species across a large environmental gradient. The inclusion of genus‐specific slopes offered a modest improvement to model performance but, more importantly, resulted in almost universally divergent environment–, trait–, and neighborhood–growth rate correlations between *Acacia* and *Eucalyptus*. This suggests restoration planning that incorporates trait‐based frameworks for multiple genera may incorrectly assume that universal trait– or neighborhood–growth relationships apply. This could occur even where combined environment–trait effects on growth, such as our moisture availability interactions here, are known and accounted for. Ultimately, while divergent correlations mapped logically onto known physiological differences between *Acacia* and *Eucalyptus*, grouping species by genus in these models does not require a priori hypotheses about physiological differences. The only assumption is that there are differences that are grouped by genus identity, which may be phylogenetically conserved at that taxonomic scale. Models like those presented here could be used to uncover genus‐mediated correlates of plant performance where detailed phylogenies are unavailable or where physiological differences are poorly understood.

Restoration ecology faces many challenges, including variation in restoration outcomes (Brudvig, 2017) that often result in failure (Suding, 2011). Rebuilding natural communities requires substantial ecological understanding (Menz et al., 2013), and trait‐based approaches that scale up restoration are essential for achieving this (e.g., Laughlin, 2014). Evolutionary and trait‐based approaches to understand plant performance are converging (Swenson et al., 2020); however, both are currently limited by data coverage (Hinchliff & Smith, 2014). It is imperative that methods be developed to improve restoration design and management that directly influence restoration outcomes. These methods need to be accessible to managers, scalable, and generalizable across ecosystems and species. Our findings suggest that taxonomic context can markedly alter expectations of plant performance even after considering environment, neighborhood, and functional traits. In lieu of true phylogenetic relationships, our results indicate that taxonomic groupings may provide a simple and accessible way to capture the complex and nuanced dynamics that exist between ecological strategies, environmental conditions, and resulting plant growth.

## CONFLICT OF INTEREST

The authors declare no conflict of interest.

## Supporting information


**Appendix S1** 

**Data S1** 

## Data Availability

Data and code (Staples, 2022) to reproduce all results and figures are available in Zenodo at https://doi.org/10.5281/zenodo.6195874. Code is also available as Supporting Information to this article as Data_S1.zip. Raw planting data used in this research were obtained from Commonwealth Scientific and Industrial Research Organization, Greening Australia, and Greenfleet via data‐sharing agreements and are not accessible to the public but may be made available to qualified researchers by contacting these organizations.
