# Supplementary material for: Drivers of Acacia and Eucalyptus growth rate differ in strength and direction in restoration plantings across Australia
Source: Ecol Appl. 2022 Jun 2;32(6):e2636. doi: 10.1002/eap.2636 (PMC9539508; doi:10.1002/eap.2636)
Supplement: Supplementary file 1 — Appendix S1 [file EAP-32-e2636-s001.pdf]

Appendix S1

Timothy L Staples, Margaret M Mayfield, Jacqueline R England, John M Dwyer
Drivers of *Acacia* and *Eucalyptus* growth rate differ in strength and direction in restoration plantings across Australia
Ecological Applications

Table S1. Focal species from neighbourhood plots, with the range of moisture availability (Ratio of total annual rainfall to total annual potential evapotranspiration) of forest plantings where the species was recorded. These values are not representations of the range of conditions the species can occur in, or that the species naturally occurs within. Trait sample sizes are the number of values for each trait used to calculate species means. Species trait means from field collection in this study are shown in Table S2.

Species	Moisture availability range	Trait sample size (n)		
		Max height	SLA	Wood density
Acacia acinacea	0.276 - 0.638	3	1	1
A. acuminata	0.217 - 0.357	2	1	4
A. argyrophylla	0.266 - 0.377	1	1	2
A. baileyana	0.377 - 0.765	3	1	1
A. brachybotrya	0.266 - 0.350	2	2	1
A. calamifolia	0.266 - 0.377	2	1	1
A. cupularis	0.306 - 0.306	2	1	1
A. cyclops	0.336 - 0.391	4	1	1
A. dealbata	0.493 - 1.210	4	11	9
A. deanei	0.357 - 0.765	2	2	1
A. decurrens	0.350 - 0.765	3	1	6
A. difformis	0.493 - 0.503	1	1	1
A. euthycarpa	0.302 - 0.312	1	1	1
A. falciformis	0.695 - 0.695	1	1	1
A. floribunda	0.512 - 0.760	2	6	1
A. hakeoides	0.266 - 0.512	4	2	1
A. implexa	0.493 - 0.765	2	4	4
A. ligulata	0.266 - 0.294	3	2	1
A. longifolia	0.385 - 0.968	7	4	2
A. maidenii	0.760 - 0.760	1	1	1
A. mearnsii	0.399 - 0.975	5	2	3
A. melanoxylon	0.505 - 1.210	6	20	10
A. microbotrya	0.217 - 0.336	1	1	1
A. microcarpa	0.278 - 0.360	2	1	1
A. montana	0.306 - 0.306	2	1	1
A. mucronata	0.573 - 0.986	1	1	1
A. notabilis	0.278 - 0.765	2	1	1
A. oswaldii	0.221 - 0.276	3	6	1
A. oxycedrus	0.618 - 0.618	2	1	1
A. paradoxa	0.317 - 0.639	3	2	1
A. penninervis	0.659 - 0.659	1	4	4
A. pravissima	0.484 - 0.760	1	1	1
A. pycnantha	0.276 - 0.888	5	1	1
A. retinodes	0.266 - 0.727	2	1	1
A. rigens	0.310 - 0.377	3	1	1
A. rubida	0.493 - 0.765	2	1	1
A. salicina	0.357 - 0.441	3	1	3
A. saligna	0.217 - 0.684	4	1	1
A. stenophylla	0.357 - 0.357	2	1	4

Species	Moisture availability range	Trait sample size (n)		
		Max height	SLA	Wood density
A. trineura	0.276 - 0.302	2	1	1
A. verniciflua	0.678 - 0.678	2	1	1
A. verticillata	0.291 - 1.210	2	1	1
A. wattsiana	0.392 - 0.392	1	1	1
Eucalyptus acaciiformis	0.727 - 0.775	1	1	1
E. albens	0.426 - 0.534	2	1	4
E. albida	0.357 - 0.357	2	1	1
E. amplifolia	0.775 - 0.786	1	9	1
E. amygdalina	0.578 - 0.839	1	1	1
E. arenacea	0.360 - 0.360	1	1	1
E. argyphea	0.357 - 0.357	1	1	1
E. astringens	0.336 - 0.357	1	1	3
E. baxteri	0.312 - 0.761	2	4	4
E. blakelyi	0.408 - 0.723	2	1	2
E. botryoides	0.509 - 0.509	5	1	4
E. brachycalyx	0.350 - 0.350	2	1	1
E. bridgesiana	0.493 - 0.724	2	1	5
E. calycogona	0.276 - 0.276	3	1	1
E. camaldulensis	0.276 - 0.726	9	3	16
E. cladocalyx	0.312 - 0.584	2	1	2
E. clivicola	0.314 - 0.314	2	1	1
E. coccifera	0.582 - 0.582	1	1	1
E. conferruminata	0.357 - 0.659	1	1	1
E. conica	0.426 - 0.426	1	1	1
E. crebra	0.441 - 0.891	2	4	7
E. dalrympleana	0.582 - 0.839	1	1	3
E. diversifolia	0.360 - 0.430	2	2	1
E. dives	0.615 - 0.765	1	2	4
E. dumosa	0.251 - 0.302	2	7	1
E. falcata	0.312 - 0.336	1	1	1
E. fasciculosa	0.302 - 0.516	1	1	1
E. gardneri	0.357 - 0.357	1	1	1
E. globulus	0.311 - 1.210	4	36	16
E. goniocalyx	0.496 - 0.765	2	2	4
E. gracilis	0.251 - 0.251	3	3	1
E. incrassata	0.221 - 0.422	2	3	1
E. kitsoniana	0.659 - 1.210	1	1	1
E. kochii	0.275 - 0.357	1	1	1
E. largiflorens	0.276 - 0.350	4	3	3
E. lehmannii	0.293 - 0.391	1	1	1
E. leptophylla	0.251 - 0.385	2	1	1
E. leucoxylon	0.266 - 0.577	5	2	3
E. longicornis	0.217 - 0.256	1	1	2
E. loxophleba	0.275 - 0.336	2	1	2
E. macrorhyncha	0.618 - 0.724	3	1	3
E. mannifera	0.496 - 0.765	2	1	1
E. megacornuta	0.311 - 0.391	1	1	1
E. melliodora	0.408 - 0.749	2	4	3
E. microcarpa	0.302 - 0.618	2	3	4
E. moluccana	0.608 - 0.891	1	1	5
E. nitens	0.484 - 0.749	1	4	5
E. obliqua	0.615 - 1.210	2	4	7
E. occidentalis	0.293 - 0.577	2	1	1
E. odorata	0.360 - 0.392	1	1	1

Species	Moisture availability range	Trait sample size (n)		
		Max height	SLA	Wood density
E. oleosa	0.276 - 0.350	6	1	2
E. ovata	0.496 - 1.210	2	1	3
E. pauciflora	0.522 - 0.786	3	3	3
E. phaenophylla	0.336 - 0.336	1	1	1
E. platypus	0.312 - 0.436	1	1	1
E. polyanthemos	0.441 - 0.758	2	1	2
E. populnea	0.441 - 0.441	1	4	5
E. porosa	0.251 - 0.360	2	1	1
E. pulchella	0.573 - 0.839	1	1	1
E. radiata	0.745 - 1.210	1	1	6
E. regnans	0.887 - 1.210	1	5	4
E. rodwayi	0.578 - 0.582	1	1	1
E. rossii	0.723 - 0.723	1	1	3
E. rubida	0.522 - 0.749	2	1	4
E. rudis	0.314 - 0.684	4	1	2
E. salubris	0.217 - 0.222	1	1	3
E. sargentii	0.217 - 0.357	2	1	1
E. siderophloia	0.891 - 0.891	1	1	4
E. sideroxylon	0.436 - 0.630	2	3	5
E. socialis	0.251 - 0.350	3	13	1
E. spathulata	0.293 - 0.336	2	1	1
E. stellulata	0.558 - 0.775	1	1	1
E. tenuiramis	0.839 - 0.839	1	1	1
E. tereticornis	0.558 - 0.891	4	1	10
E. tricarpa	0.436 - 0.659	2	1	1
E. utilis	0.311 - 0.391	2	1	1
E. viminalis	0.360 - 1.210	4	4	4
E. viridis	0.505 - 0.505	2	2	1
E. wandoo	0.256 - 0.357	3	1	3
E. willisii	0.740 - 0.888	1	1	1

Table S2. Trait values for species collected in this study. Only specific leaf area (SLA) and wood density traits were estimated from field samples, the rest were obtained from existing sources. Primarily, seed mass values were obtained from Royal Botanical Gardens (KEW), and maximum height values were obtained from Australian State herbarium records: Florabase, eFlora and Plantnet. Other SLA and wood density estimates were obtained through the TRY Plant Trait Database (see main text for TRY sources). Values are presented to 5 decimal places, and were estimated as per recommendations in Perez-Harguindeguy et al. (2013) (one sample from five individuals for wood density, and 5 samples from 5 individuals for SLA).

Species	SLA ($mm^2\ g^{-1}$)	Wood density ($g\ cm^{-3}$)
Acacia acinacea	6.74126	0.77414
Acacia baileyana	8.64803	0.74308
Acacia calamifolia	3.10424	0.82043
Acacia cupularis	3.37138	0.60801
Acacia difformis	5.46410	0.71673
Acacia euthycarpa	2.64535	0.90105
Acacia falciformis	5.64041	0.64357
Acacia microbotrya	4.9638	0.73062
Acacia microcarpa	2.68416	0.82679
Acacia montana	6.73812	0.81742
Acacia mucronata	4.87214	0.56005
Acacia notabilis	4.77474	0.84122
Acacia oxycedrus	2.64889	0.64460
Acacia pravissima	8.01686	0.58749
Acacia pycnantha	4.26198	0.62091
Acacia retinodes	7.58876	0.74884
Acacia verniciflua	6.40996	0.78483
Acacia verticillata	5.86454	0.62655
Acacia wattsiana	4.60890	0.71820
Eucalyptus acaciiformis	5.34734	0.61311
Eucalyptus argyphea	2.96700	0.86063
Eucalyptus brachycalyx	3.05878	0.86829
Eucalyptus clivicola	2.70495	0.84124
Eucalyptus coccifera	3.63351	0.60466
Eucalyptus conferruminata	3.56764	0.78434
Eucalyptus falcata	2.78543	0.83092
Eucalyptus fasciculosa	4.13049	0.64471
Eucalyptus gardneri	3.50454	0.82013
Eucalyptus kitsoniana	5.84300	0.57247
Eucalyptus kochii	3.10828	0.81919
Eucalyptus lehmannii	2.77751	0.90510
Eucalyptus longicornis	3.15267	0.90205
Eucalyptus megacornuta	3.80134	0.80936
Eucalyptus occidentalis	2.88290	0.73930
Eucalyptus odorata	3.76189	0.84655
Eucalyptus phaenophylla	2.60989	0.86959
Eucalyptus platypus	2.43897	0.88758
Eucalyptus porosa	4.06599	0.71928
Eucalyptus pulchella	4.03020	0.71428
Eucalyptus rodwayi	4.46687	0.58843
Eucalyptus sargentii	3.41637	0.84431
Eucalyptus spathulata	3.58704	0.87205
Eucalyptus stellulata	4.33103	0.57299
Eucalyptus tenuiramis	4.12743	0.70121
Eucalyptus tricarpa	3.83635	0.71786
Eucalyptus utilis	2.53216	0.85996
Eucalyptus willisii	3.92239	0.54456

Tables S3-7 are linear mixed-effects model summaries, testing for differences in the effect of climate, functional traits and competition on the growth rate of *Eucalyptus* and *Acacia* species. Genus was a two-level factor, with Acacia set as the reference level.

Table S3. Intercept only model ("Null model").

Variable	Estimate	Std. Error	*t*-value
Intercept	0.256	0.015	16.531

Random effects	Variance
Among IBRA sub-regions	0.004
Among plantings, within IBRA sub-regions	0.010
Among plots within plantings	0.001
Residual	0.024

Table S4. Model excluding genus, containing all theorized predictors of growth rate ("No genus model").

Variable	Estimate	Std. Error	*t*-value
Intercept	0.233	0.014	17.114
Moisture availability	-0.002	0.014	-0.164
Solar radiation	-0.014	0.014	-0.984
Specific leaf area	0.004	0.002	2.155
Wood density	0.007	0.002	3.349
Maximum height	-0.001	0.002	-0.605
Planting age	-0.070	0.006	-11.157
Neighbor density	-0.043	0.004	-11.849
Proportion intraspecific neighbors	-0.025	0.002	-14.021
Plot area	0.010	0.007	1.533
Neighborhood richness	-0.004	0.005	-0.760
Functional evenness	0.001	0.003	0.393
Functional divergence	-0.006	0.004	-1.629
Plot area:Proportion intraspecific neighbors	-0.016	0.002	-9.515

Random effects	Variance
Between IBRA sub-regions	0.005
Between plantings within IBRA sub-regions	0.003
Between plots within plantings	0.005
Residual	0.024

Table S5. Model including genus, containing all theorized predictors of growth rate and fit with genus as a two-level factor interacting with all other model terms ("Genus slopes model").

Variable	Estimate	Std. Error	*t*-value
Intercept	0.131	0.014	9.124
Moisture availability	0.001	0.013	0.050
Solar radiation	-0.035	0.014	-2.601
Specific leaf area	0.041	0.002	16.462
Wood density	0.032	0.004	8.931
Maximum height	-0.054	0.005	-11.849
Planting age	-0.107	0.007	-16.268
Neighbor density	-0.045	0.005	-9.980
Proportion intraspecific neighbors	-0.039	0.003	-13.338
Plot area	-0.012	0.007	-1.795
Neighborhood richness	0.022	0.006	3.655
Functional evenness	0.007	0.004	1.710
Functional divergence	-0.010	0.005	-2.046
Genus (Eucalyptus)	0.139	0.007	19.307
Plot area:Proportion intraspecific neighbors	-0.033	0.002	-13.297
Moisture availability:Eucalyptus	-0.010	0.005	-2.015
Solar radiation:Eucalyptus	0.032	0.005	6.076
Specific leaf area:Eucalyptus	-0.020	0.006	-3.160
Wood density:Eucalyptus	-0.051	0.004	-11.527
Maximum height:Eucalyptus	0.044	0.005	8.144
Planting age:Eucalyptus	0.043	0.004	10.147
Neighbor density:Eucalyptus	0.010	0.004	2.843
Proportion intraspecific neighbors:Eucalyptus	0.044	0.004	10.777
Plot area:Eucalyptus	0.036	0.004	8.931
Neighborhood richness:Eucalyptus	-0.026	0.005	-4.995
Functional evenness:Eucalyptus	-0.009	0.004	-2.231
Functional divergence:Eucalyptus	0.002	0.004	0.449
Plot area:Proportion intraspecific neighbors:Eucalyptus	0.044	0.004	12.079

Random effects	Variance
Between IBRA sub-regions	0.005
Between plantings within IBRA sub-regions	0.003
Between plots within plantings	0.005
Residual	0.024

Table S6. Model excluding genus with moisture availability interactions. Containing all theorized predictors of growth rate fit with two-way interaction terms with moisture availability ("No genus moisture model").

Variable	Estimate	Std. Error	*t*-value
Intercept	0.232	0.015	15.027
Moisture availability	-0.003	0.014	-0.221
Solar radiation	-0.014	0.014	-1.014
Specific leaf area	0.005	0.002	3.184
Wood density	0.006	0.002	2.556
Maximum height	-0.001	0.002	-0.566
Planting age	-0.070	0.007	-10.530
Neighbor density	-0.045	0.004	-11.663
Proportion intraspecific neighbors	-0.024	0.002	-13.305
Plot area	0.013	0.007	1.831
Neighborhood richness	-0.003	0.005	-0.564
Functional evenness	0.001	0.003	0.418
Functional divergence	-0.005	0.004	-1.442
Plot area:Proportion intraspecific neighbors	-0.014	0.002	-8.196
Solar radiation:Moisture availability	-0.001	0.010	-0.149
Specific leaf area:Moisture availability	-0.001	0.002	-0.357
Wood density:Moisture availability	-0.005	0.002	-1.849
Maximum height:Moisture availability	-0.001	0.002	-0.322
Planting age:Moisture availability	0.005	0.007	0.779
Neighbor density:Moisture availability	-0.006	0.004	-1.546
Proportion intraspecific neighbors:Moisture availability	-0.012	0.002	-6.017
Plot area:Moisture availability	0.002	0.008	0.203
Neighborhood richness:Moisture availability	-0.001	0.005	-0.139
Functional evenness:Moisture availability	0.005	0.002	2.010
Functional divergence:Moisture availability	-0.001	0.003	-0.434
Plot area:Proportion intraspecific neighbors:Moisture availability	-0.025	0.002	-11.777

Random effects	Variance
Between IBRA sub-regions	0.004
Between plantings within IBRA sub-regions	0.003
Between plots within plantings	0.001
Residual	0.024

Table S7. Model including genus with moisture availability interactions. Containing all theorized predictors of growth rate fit with interaction terms with moisture availability ("Genus slopes moisture model").

Variable	Estimate	Std. Error	*t*-value
Intercept	0.114	0.018	6.457
Moisture availability	-0.032	0.017	-1.896
Solar radiation	-0.047	0.014	-3.424
Specific leaf area	0.057	0.003	18.151
Wood density	0.036	0.006	5.830
Maximum height	-0.081	0.006	-14.543
Planting age	-0.109	0.007	-15.305
Neighbor density	-0.049	0.005	-10.590
Proportion intraspecific neighbors	-0.034	0.003	-10.625
Plot area	-0.007	0.008	-0.893
Neighborhood richness	0.023	0.006	3.587
Functional evenness	0.008	0.004	2.107
Functional divergence	-0.010	0.005	-2.061
Genus (Eucalyptus)	0.140	0.011	13.205
Plot area:Proportion intraspecific neighbors	-0.028	0.003	-10.667
Moisture availability:Eucalyptus	0.023	0.011	2.067
Solar radiation:Eucalyptus	0.048	0.006	8.701
Specific leaf area:Eucalyptus	-0.036	0.008	-4.708
Wood density:Eucalyptus	-0.060	0.007	-8.975
Maximum height:Eucalyptus	0.076	0.006	11.945
Planting age:Eucalyptus	0.048	0.004	10.948
Neighbor density:Eucalyptus	0.009	0.004	2.595
Proportion intraspecific neighbors:Eucalyptus	0.035	0.004	8.136
Plot area:Eucalyptus	0.029	0.004	6.620
Neighborhood richness:Eucalyptus	-0.023	0.005	-4.270
Functional evenness:Eucalyptus	-0.010	0.004	-2.394
Functional divergence:Eucalyptus	0.003	0.005	0.553
Solar radiation:Moisture availability	0.020	0.010	1.920
Specific leaf area:Moisture availability	0.035	0.003	10.256
Wood density:Moisture availability	0.017	0.006	2.889
Maximum height:Moisture availability	-0.028	0.005	-5.025
Planting age:Moisture availability	-0.011	0.007	-1.508
Neighbor density:Moisture availability	-0.019	0.005	-3.807
Proportion intraspecific neighbors:Moisture availability	0.009	0.004	2.276
Plot area:Moisture availability	0.019	0.008	2.263
Neighborhood richness:Moisture availability	0.021	0.007	3.193
Functional evenness:Moisture availability	0.010	0.004	2.857
Functional divergence:Moisture availability	-0.011	0.005	-2.154
Plot area:Proportion intraspecific neighbors:Eucalyptus	0.040	0.004	10.516
Plot area:Proportion intraspecific neighbors:Moisture availability	-0.006	0.004	-1.620
Solar radiation:Moisture availability:Eucalyptus	-0.034	0.005	-6.430
Specific leaf area:Moisture availability:Eucalyptus	-0.029	0.007	-4.371
Wood density:Moisture availability:Eucalyptus	-0.026	0.007	-3.921
Maximum height:Moisture availability:Eucalyptus	0.027	0.006	4.515
Planting age:Moisture availability:Eucalyptus	0.029	0.005	5.774
Neighbor density:Moisture availability:Eucalyptus	0.013	0.004	3.071
Proportion intraspecific neighbors:Moisture availability:Eucalyptus	-0.008	0.005	-1.512
Plot area:Moisture availability:Eucalyptus	-0.017	0.005	-3.315
Neighborhood richness:Moisture availability:Eucalyptus	-0.024	0.006	-3.748
Functional evenness:Moisture availability:Eucalyptus	-0.009	0.004	-2.374
Functional divergence:Moisture availability:Eucalyptus	0.007	0.005	1.594

Plot area:Proportion intraspecific neighbors:Moisture availability:Eucalyptus	-0.001	0.005	-0.249

Random effects	Variance
Between IBRA sub-regions	0.004
Between plantings within IBRA sub-regions	0.003
Between plots within plantings	0.001
Residual	0.022

Table S8: Comparison of observed and expected slopes of genus model of *Acacia* and *Eucalyptus* growth rate. Expected distribution was established by modelling data 1,000 times, with growth rates randomly swapped between focal plants across the dataset. The *p*-values here refer to the position of the observed slope relative to the distribution of null values (*p* for values less than the null distribution mean, and 1-*p* for values greater than the mean). The critical threshold for these *p*-values is therefore 0.025 (not 0.05), reflecting that the observed slope lies outside the 95% quantiles of the null distribution.

Genus	Fixed effect	Observed slope	2.5% expected CI	97.5% expected CI	*p*-value
Acacia	Moisture availability	-0.003	-0.008	0.008	0.194
Acacia	Solar radiation	-0.035	-0.007	0.008	< 0.001
Acacia	SLA	0.027	-0.005	0.005	< 0.001
Acacia	Wood density	0.013	-0.007	0.007	< 0.001
Acacia	Maximum height	-0.063	-0.009	0.009	< 0.001
Acacia	Planting age	-0.103	-0.006	0.006	< 0.001
Acacia	Planting density	-0.053	-0.006	0.006	< 0.001
Acacia	Proportion intraspecific neighbours	-0.022	-0.006	0.006	< 0.001
Acacia	Plot area	-0.005	-0.005	0.005	0.033
Acacia	Neighborhood richness	0.014	-0.008	0.008	< 0.001
Acacia	Functional evenness	0.009	-0.006	0.006	0.003
Acacia	Functional dispersion	-0.010	-0.006	0.007	0.001
Eucalyptus	Moisture availability	-0.007	-0.006	0.007	0.022
Eucalyptus	Solar radiation	-0.004	-0.006	0.006	0.089
Eucalyptus	SLA	-0.004	-0.011	0.011	0.268
Eucalyptus	Wood density	-0.004	-0.005	0.005	0.053
Eucalyptus	Maximum height	0.006	-0.006	0.006	0.039
Eucalyptus	Planting age	-0.063	-0.004	0.004	< 0.001
Eucalyptus	Planting density	-0.033	-0.004	0.004	< 0.001
Eucalyptus	Proportion intraspecific neighbours	-0.002	-0.005	0.005	0.175
Eucalyptus	Plot area	0.011	-0.005	0.005	< 0.001
Eucalyptus	Neighborhood richness	0.000	-0.006	0.006	0.466
Eucalyptus	Functional evenness	-0.003	-0.004	0.004	0.081
Eucalyptus	Functional dispersion	-0.011	-0.005	0.005	< 0.001

Table S9: Comparison of observed and expected slopes of genus moisture model of *Acacia* and *Eucalyptus* growth rate. Expected distribution was established by modelling data 1,000 times, with growth rates randomly swapped between focal plants across the dataset. Dry and mesic conditions were the 10th and 90th quantile of moisture availability in the dataset: 0.27 and 076 respectively. The *p*-values here refer to the position of the observed slope relative to the distribution of null values (*p* for values less than the null distribution mean, and 1-*p* for values greater than the mean). The critical threshold for these *p*-values is therefore 0.025 (not 0.05), reflecting that the observed slope lies outside the 95% quantiles of the null distribution.

Genus	Fixed effect	Conditions	Observed slope	2.5% expected CI	97.5% expected CI	*p*-value
Acacia	Solar radiation	Dry	-0.069	-0.013	0.013	< 0.001
Acacia	Solar radiation	Mesic	0.014	-0.015	0.016	0.041
Acacia	Planting age	Dry	-0.080	-0.010	0.010	< 0.001
Acacia	Planting age	Mesic	-0.153	-0.014	0.014	< 0.001
Acacia	Plant density	Dry	-0.024	-0.010	0.010	< 0.001
Acacia	Plant density	Mesic	-0.087	-0.013	0.013	< 0.001
Acacia	SLA	Dry	0.026	-0.007	0.007	< 0.001
Acacia	SLA	Mesic	0.051	-0.015	0.016	< 0.001
Acacia	Wood density	Dry	0.021	-0.009	0.009	< 0.001
Acacia	Wood density	Mesic	-0.104	-0.031	0.029	< 0.001
Acacia	Maximum height	Dry	-0.083	-0.014	0.014	< 0.001
Acacia	Maximum height	Mesic	-0.081	-0.025	0.025	< 0.001
Acacia	Neighborhood richness	Dry	0.002	-0.014	0.014	0.357
Acacia	Neighborhood richness	Mesic	0.025	-0.020	0.019	0.005
Acacia	Functional evenness	Dry	0.000	-0.011	0.011	0.484
Acacia	Functional evenness	Mesic	0.024	-0.010	0.010	< 0.001
Acacia	Functional dispersion	Dry	-0.003	-0.010	0.011	0.256
Acacia	Functional dispersion	Mesic	-0.017	-0.014	0.014	0.011
Acacia	Proportion intraspecific neighbours	Dry	-0.043	-0.012	0.012	< 0.001
Acacia	Proportion intraspecific neighbours	Mesic	0.006	-0.011	0.011	0.135
Acacia	Plot area	Dry	-0.036	-0.009	0.010	< 0.001
Acacia	Plot area	Mesic	0.006	-0.011	0.011	0.135
Eucalyptus	Solar radiation	Dry	0.016	-0.010	0.010	0.001
Eucalyptus	Solar radiation	Mesic	-0.023	-0.010	0.010	< 0.001
Eucalyptus	Planting age	Dry	-0.082	-0.006	0.006	< 0.001
Eucalyptus	Planting age	Mesic	-0.035	-0.011	0.011	< 0.001
Eucalyptus	Plant density	Dry	-0.036	-0.006	0.006	< 0.001
Eucalyptus	Plant density	Mesic	-0.040	-0.007	0.007	< 0.001
Eucalyptus	SLA	Dry	-0.022	-0.023	0.024	0.034
Eucalyptus	SLA	Mesic	0.042	-0.018	0.017	< 0.001
Eucalyptus	Wood density	Dry	0.007	-0.008	0.008	0.052
Eucalyptus	Wood density	Mesic	-0.044	-0.012	0.012	< 0.001
Eucalyptus	Maximum height	Dry	0.026	-0.009	0.008	< 0.001
Eucalyptus	Maximum height	Mesic	-0.052	-0.013	0.013	< 0.001
Eucalyptus	Neighborhood richness	Dry	0.003	-0.010	0.009	0.240
Eucalyptus	Neighborhood richness	Mesic	-0.002	-0.013	0.013	0.363
Eucalyptus	Functional evenness	Dry	-0.004	-0.007	0.007	0.107

Eucalyptus	Functional evenness	Mesic	-0.001	-0.005	0.005	0.421
Eucalyptus	Functional dispersion	Dry	-0.003	-0.007	0.007	0.167
Eucalyptus	Functional dispersion	Mesic	-0.013	-0.009	0.009	0.002
Eucalyptus	Proportion intraspecific neighbours	Dry	-0.008	-0.008	0.009	0.033
Eucalyptus	Proportion intraspecific neighbours	Mesic	-0.007	-0.011	0.011	0.106
Eucalyptus	Plot area	Dry	0.010	-0.007	0.007	0.001
Eucalyptus	Plot area	Mesic	-0.007	-0.011	0.011	0.106

Table S10. Linear mixed-effects model summary estimating the effect of planting age, moisture availability and species-level density on total above-ground biomass of *Acacia* plants.

Fixed effects	Estimate	Std. Error	*t*-value
Intercept	2.055	0.141	14.524
ln(Planting age)	0.205	0.091	2.245
Moisture availability	0.365	0.127	2.882
Conspecific density	-0.589	0.039	-14.923
Heterospecific density	-0.059	0.043	-1.368

Random effects	Variance
Among IBRA sub-regions	0.341
Among plantings, within IBRA sub-regions	0.400
Among plots, within plantings	0.147
Residual	2.276

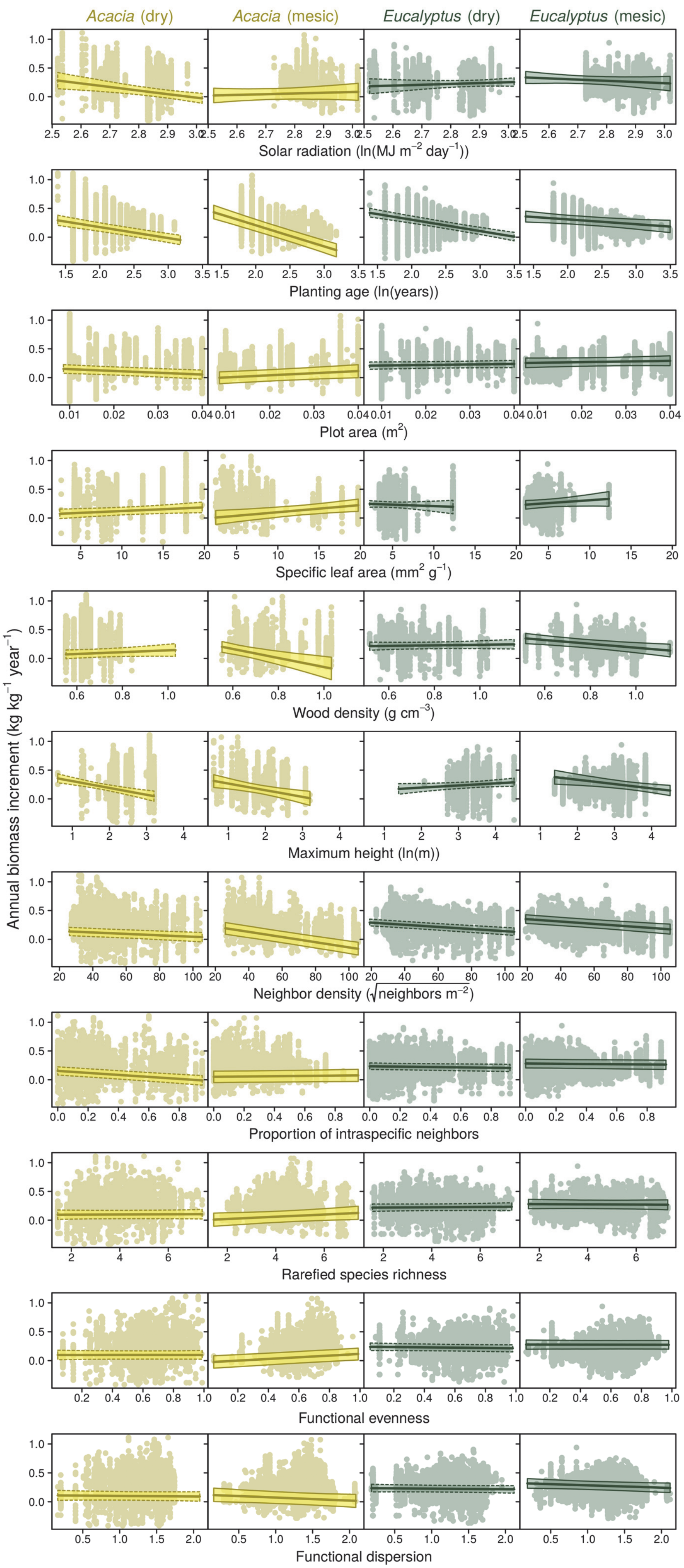

Figure S1: Partial regression slopes from the "genus IS moisture model". Each row is a predictor variable (see x-axis labels), each column represents either *Acacia* or *Eucalyptus* slopes in dry or mesic conditions. Dry and mesic conditions are the 10th and 90th quantile of moisture availability in the dataset, 0.27 and 0.76 respectively. Given that these slopes are part of a continuous interaction effect, rather than discrete slopes applied to different sets of data, we have presented raw data points below the mean of each variable in the dry subplots, and points above the mean in the mesic subplots. These raw data do not account for the effect of other predictors or random effects.

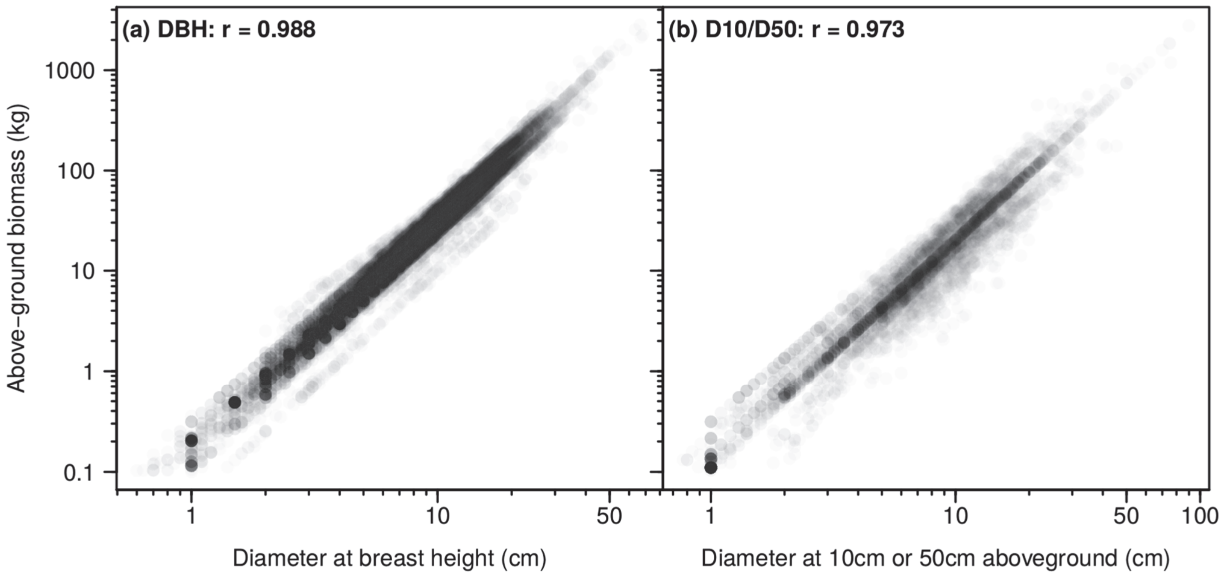

Figure S2: Correlation of above-ground biomass estimates derived from allometric equations, and diameter measurements taken during plot surveys. (a) shows diameter at breast height measurements, and (b) shows an aggregate of diameter measurements taken at either 10cm or 50cm above-ground (measurements were not distinguished in the raw data).

Supplementary References

Perez-Harguindeguy, N. et al. (2013). New handbook for standardised measurement of plant functional traits worldwide, Australian Journal of Botany, 61, 167-234.
