# Supplementary material for: Drivers of Acacia and Eucalyptus growth rate differ in strength and direction in restoration plantings across Australia
Source: Ecol Appl. 2022 Jun 2;32(6):e2636. doi: 10.1002/eap.2636 (PMC9539508; doi:10.1002/eap.2636)
Supplement: Supplementary file 2 — Data S1 [file EAP-32-e2636-s002.zip › Metadata_S1.pdf]

Timothy L Staples, Margaret M Mayfield, Jacqueline R England, John M Dwyer. 2022. Drivers of *Acacia* and *Eucalyptus* growth rate differ in strength and direction in restoration plantings across Australia. *Ecological Applications*

Data S1

R script used to process restoration planting inventory data, run analyses and produce figures. Data and code to reproduce results are hosted on Zenodo at https://doi.org/10.5281/zenodo.6195874

Author of the material provided in Data_S1.zip

Timothy L Staples
School of Biological Sciences, The University of Queensland, Brisbane, Queensland, Australia, 4072
timothy.staples@uqconnect.edu.au

File list (file found within DataS1.zip)

`R-script-for-data-processing-analysis-and-figures.R`

Description

`R-script-for-data-processing-analysis-and-figures.R` – R script used to process restoration planting inventory data, run analyses and produce figures.
